# Dysregulated thrombospondin 1 and miRNA-29a-3p in severe COVID-19

**DOI:** 10.1038/s41598-022-23533-x

**Published:** 2022-12-08

**Authors:** In Soo Kim, Sung-Gwon Lee, Seul Gi Shin, Hyeongseok Jeong, Kyung Mok Sohn, Ki-Sun Park, Prashanta Silwal, Shinhye Cheon, Jungok Kim, Sungmin Kym, Yeon-Sook Kim, Eun-Kyeong Jo, Chungoo Park

**Affiliations:** 1 Department of Medical Science, Chungnam National University School of Medicine, Daejeon, Korea; 2 Department of Microbiology, Chungnam National University School of Medicine, Daejeon, Korea; 3 Infection Control Convergence Research Center, Chungnam National University School of Medicine, Daejeon, Korea; 4 School of Biological Sciences and Technology, Chonnam National University, Gwangju, Korea; 5 Division of Infectious Diseases, Department of Internal Medicine, Chungnam National University School of Medicine, Daejeon, Korea; 6 KM Science Research Division, Korea Institute of Oriental Medicine, Daejeon, Korea

**Keywords:** High-throughput screening, Cellular signalling networks, Viral infection

## Abstract

Although nearly a fifth of symptomatic COVID-19 patients suffers from severe pulmonary inflammation, the mechanism of developing severe illness is not yet fully understood. To identify significantly altered genes in severe COVID-19, we generated messenger RNA and micro-RNA profiling data of peripheral blood mononuclear cells (PBMCs) from five COVID-19 patients (2 severe and 3 mild patients) and three healthy controls (HC). For further evaluation, two publicly available RNA-Seq datasets (GSE157103 and GSE152418) and one single-cell RNA-Seq dataset (GSE174072) were employed. Based on RNA-Seq datasets, thrombospondin 1 (THBS1) and interleukin-17 receptor A (IL17RA) were significantly upregulated in severe COVID-19 patients’ blood. From single-cell RNA-sequencing data, *IL17RA* level is increased in monocytes and neutrophils, whereas *THBS1* level is mainly increased in the platelets. Moreover, we identified three differentially expressed microRNAs in severe COVID-19 using micro-RNA sequencings. Intriguingly, *hsa-miR-29a-3p* significantly downregulated in severe COVID-19 was predicted to bind the 3′-untranslated regions of both *IL17RA* and *THBS1* mRNAs. Further validation analysis of our cohort (8 HC, 7 severe and 8 mild patients) showed that *THBS1*, but not *IL17RA*, was significantly upregulated, whereas *hsa-miR-29a-3p* was downregulated, in PBMCs from severe patients. These findings strongly suggest that dysregulated expression of *THBS1*, *IL17RA*, and *hsa-miR-29a-3p* involves severe COVID-19.

## Introduction

Coronavirus disease 2019 (COVID-19), caused by the severe acute respiratory syndrome coronavirus 2 (SARS-CoV-2), is a serious threat to global public health. As per the WHO living guidance for COVID-19 management, 15% of symptomatic COVID-19 patients develop severe disease characterized by respiratory distress, and 5% are critically ill^1,2^. So far, much effort has been made to comprehend the clinical, biological, and immune characteristics associated with the severity of COVID-19. Recent meta-analysis studies found that serum C-reactive protein, lactate dehydrogenase, and D-dimer levels are significantly linked with the severity of COVID-19^3,4^. In addition, older age, male sex, and comorbidity have been identified as the risk factors for critical illness and death caused by SARS-CoV-2^5–7^. Severe patients admitted to the intensive care unit typically hospitalize longer, are more likely to require mechanical ventilation due to respiratory failure, and have higher mortality^8,9^. To effectively control the poor outcomes, it is important to understand a mechanism for developing severe COVID-19.

Multiple studies on severe COVID-19 have highlighted immunological perturbations such as reduced T cell subsets, increased neutrophil to lymphocyte ratio, and elevated proinflammatory cytokine production^10–12^. Proinflammatory cytokine overexpression contributes to pulmonary inflammation and pathological lung damage in COVID-19 patients with cytokine release syndrome^13,14^. Interleukin (IL)-17 is one of the proinflammatory cytokines released from patients with COVID-19-related cytokine release syndrome^15,16^. In addition, thrombosis is a deadly complication of respiratory virus infections, including COVID-19. The crosstalks between coagulation and immune system may determine the severity of pulmonary pathology during viral infections^17,18^. MicroRNA (miRNA) is a small non-coding RNA and is widely studied as a biomarker and regulator of numerous human diseases^19,20^. Recent studies have attempted to figure out miRNA profiles which correlate with clinical severity in peripheral blood from COVID-19 patients^21–23^. Despite this, the actual predicting markers of severe COVID-19 remained obscure until recently. Identifying effective targets associated with clinical severity will develop new preventive and therapeutic strategies against COVID-19. Therefore, this study aimed to investigate the transcriptomic characteristics correlated with the severe COVID-19.

We previously showed no significant difference between severe and mild/moderate COVID-19 cases in the context of immune-related transcriptomic profiles by the nCounter Human Immunology gene expression assay^24^. However, for unbiased screening of a whole range of transcripts between mild and severe COVID-19 patients, we sequenced total messenger and small RNAs from peripheral blood mononuclear cells (PBMCs) of COVID-19 patients (three mild and two severe illnesses) and three healthy controls (HC). Our sequencing data, as well as publicly available two RNA sequencing (RNA-Seq) and one single-cell RNA-Seq data, revealed that both IL-17 receptor A (IL17RA) and thrombospondin 1 (THBS1) mRNA levels were elevated, but the *hsa-miR-29a-3p* level was down-regulated, in PBMCs from the severe group, when compared to those from HCs and mild group of COVID-19. Further experimental analysis of our cohort (8 HCs, 7 severe and 8 mild patients) showed that *THBS1*, but not *IL17RA*, was significantly upregulated, whereas *hsa-miR-29a-3p* was downregulated, in PBMCs from severe patients compared with those from HCs. These findings contribute to laying the groundwork for developing novel therapeutic strategies for severe COVID-19.

## Methods

### Patients and samples

A real-time quantitative polymerase chain reaction was used to establish the presence of SARS-CoV-2 in the nasopharyngeal and oropharyngeal swabs or sputum of COVID-19 patients. Mild and severe patients were classified into ‘0–1’ and ‘6–7’, respectively, depending on the WHO severity score^1^. The study included COVID-19 patients hospitalized at Chungnam National University Hospital, and all subjects were given informed consent including age/sex-matched HCs. Patients under the age of 19 were excluded. All clinical and laboratory findings were at the time when the samples were taken.

### Sample preparation and total RNA extraction

PBMCs from heparinized venous blood was isolated using a density gradient medium, Lymphoprep (STEMCELL Technologies, Vancouver, Canada), as detailed previously^24^. Total RNA from PBMCs was isolated with QIAzol lysis reagent (Qiagen, Hilden, Germany) and miRNeasy Mini Kits (Qiagen) according to the manufacturer’s instructions. RNA quality was evaluated using Agilent 2100 bioanalyzer with the RNA 6000 Pico Chip (Agilent Technologies, CA, USA). RNA was quantified using a NanoDrop 2000 Spectrophotometer system (Thermo Fisher Scientific, MA, USA).

### Library preparation and sequencing

Regarding RNA-sequencing (RNA-Seq), QuantSeq 3′ mRNA-Seq Library Prep Kit (Lexogen, Wien, Austria) was used for the library construction. In brief, reverse transcription was performed with each 500 ng total RNA after hybridization using an oligo-dT primer linked with an Illumina-compatible sequence at 5′ end. Next to RNA template degradation, the second strand was synthesized with random primers containing an Illumina-compatible linker sequence at its 5′ end. All reaction components were removed using magnetic beads purifying the double-stranded library. The complete adapter sequences for cluster generation were added by amplifying the library. After purification of the finished library, single-end 75 sequencings were performed with NextSeq 500 (Illumina, CA, USA).

For small RNA-sequencing (smRNA-Seq), the NEBNext Multiplex Small RNA Library Prep kit (New England BioLabs, MA, USA) was used for the construction of the library. Shortly, after ligation adaptors to each 1 μg total RNA, reverse transcription was performed with adaptor-specific primers. The library was amplified and purified using QIAquick PCR Purification Kit (Qiagen) and AMPure XP beads (Beckman Coulter, CA, USA). The yield and size distribution of the small RNA libraries was assessed using Agilent 2100 Bioanalyzer instrument for the High-sensitivity DNA Assay (Agilent Technologies). Single-end 75 sequencings were performed by the NextSeq500 system (Illumina).

### RNA-Seq analysis

To remove low-quality bases (< Q20), all raw sequence reads were fed to BBduk, a tool of BBMap package (https://sourceforge.net/projects/bbmap). Next, remained reads from QuantSeq 3′ mRNA-Seq and small RNA-Seq were mapped to the human reference genome (GRCh37/hg19)^25^ and mature miRNA sequences of miRBase database^26^ using Bowtie2 software^27^, respectively. We calculated read counts of genes with Bedtools^28^ and performed quantile normalization using EdgeR^29^. Unless otherwise stated, the unit of expression level in our analyses is quantile normalized read count. For identifying differentially expressed genes (DEGs), gene expression levels between groups were analyzed statistically by applying Student’s *t* test recommended by the protocol, and we defined DEGs with p-value < 0.05 and twofold change. To characterize the genes responsible for the COVID-19 disease, we analyzed the enrichment of gene ontology (GO) using the Database for Annotation, Visualization, and Integrated Discovery (DAVID, http://david.abcc.ncifcrf.gov). The expression heatmap of DEGs was depicted using R pheatmap package (version 1.0.12). Protein interaction relationships were analyzed using STRING protein interaction database (version 11)^30^.

### Meta-data analysis of publicly available RNA-sequencing data

Three COVID-19-related publicly available RNA-sequence datasets were retrieved from the NCBI GEO database^31^. The first cohort data^32^ (GSE157103) was studied for peripheral blood leukocyte transcriptome from 100 COVID-19-positive patients. We discriminated 42 severe and 58 moderate cases of COVID-19-positive patients by the use of mechanical ventilator support. The second cohort data^33^ (GSE152418) contained PBMC transcriptomes from 16 acute COVID-19 hospitalized patients, consisting of 8 severe, 4 ICU, and 4 moderate patients. Twelve patients labeled with severe or ICU were considered the severe case, and four patients labeled moderate were grouped as the moderate case. From each obtained quantified transcriptome expression data, the edgeR R-package (version 3.36.0)^29^ was used for transformation of the raw counts into counts per million (CPM) and for exclusion of very lowly expressed genes. Genes with log2-CPM ≥ 1 in at least 2 samples were kept for further analysis. After filtering, to scale the raw library sizes, normalization factors were calculated with the trimmed mean of M-values (TMM) method using the calcNormFactors function in edgeR of the R package (version 3.36.0)^29^. Differential expression analysis was performed using the glmFit and glmLRT functions embedded in the edgeR package. The false discovery rate (FDR) of Benjamini and Hochberg was used to correct for multiple testing, and only genes with FDR < 0.05 and a 1.5-fold change cutoff were considered significantly differentially expressed.

In the third cohort data^34^ (GSE174072), there were RBC-lysed whole blood single-cell RNA-sequencing (scRNA-Seq) datasets of 41 samples, including 33 COVID-19 patients and 8 healthy controls. We obtained their processed scRNA-Seq data from the COVID-19 Cell Altas (https://www.covid19cellatlas.org/). To identify genes differentially expressed among the cell populations, we used the ‘FindAllMarkers’ function in Seurat (version 4.0.5)^35^ using default parameters. Statistical significance was determined by Seurat’s implementation of the two-sided Wilcoxon rank-sum test with Bonferroni’s correction.

### MiRNA target gene prediction

Putative target genes and binding sites of the indicated microRNAs (miRNAs) were predicted using miRWalk 3.0 online prediction software (http://mirwalk.umm.uni-heidelberg.de/; last accessed February 2022)^36^. Minimum free energy (ΔG) for each miRNA-target pair was also calculated by miRWalk 3.0.

### Cell culture and transfections

THP-1 cells were purchased from American Type Culture Collection (TIB-202, ATCC, VA, USA). THP-1 cells were maintained in a humidified incubator at 37 °C temperature and 5% CO_2_ conditions in RPMI 1640 media (12-702F, Lonza, Basel, Switzerland) supplemented with 10% FBS and 1% penicillin/streptomycin. For transfection, THP-1 cells were seeded at 3 × 10^5^ per well in 48-well plates and differentiated for 3 h with Phorbol-12-myristate-13-acetate (P8139, Sigma-Aldrich, MO, USA) of 500 nM concentration. After 3 h, THP-1 cells were transfected with mimic negative control (50 nM) or *hsa-miR-29a-3p* (5, 20, 50 nM) using Lipofectamine 2000 (12566014, Invitrogen, MA, USA) according to the manufacturer’s instructions. The *hsa-miR-29a-3p* mimic (5′-UAGCACCAUCUGAAAUCGGUUA-3′) was purchased from Genolution (Seoul, South Korea). The mimic negative control was purchased from Ambion (4464058, TX, Austin).

### Quantitative real-time polymerase chain reaction analysis (qPCR)

After total RNA extraction, complementary DNA was synthesized using a reverse transcription master mix (EBT-1515C, Elpis Biotech, London, England) for mRNA expression analysis, and miScript II RT kits (218161, Qiagen) for miRNA expression analysis. qPCR was performed in the Rotor-Gen Q 2plex system (9001620, Qiagen) using the Quantinova SYBR Green PCR Kit (208056, Qiagen) or miScript SYBR Green PCR Kit (218073, Qiagen). Data were analyzed using the delta-delta CT relative quantification method with human *ACTIN* or *RNU6-2* as an internal control gene. Primer sequences were as follows: *ACTIN* forward: 5′-CACCATTGGCAATGAGCGGTTC-3′, reverse: 5′-AGGTCTTTGCGGATGTCCACGT-3′, *IL17RA* forward: 5′-AGTTCCACCAGCGATCCAAC-3′, reverse: 5′-GGCATGTAGTCCGGAATTGG-3′, *THBS1* forward: 5′-CAGGGATACTCGGGCCTTTC-3′, reverse: 5′-GAAACCCGTCTTTGGCCTGT-3′, *Hsa-miR-29a-3p*: 5′-TAGCACCATCTGAAATCGGTTA-3′. Primer for *RNU6-2* was purchased from Qiagen (MS00033740).

### Ethics statement

This study was approved by the Institutional Research and Ethics Committee at Chungnam National University Hospital (Daejeon, Korea; CNUH 2019-04-046, CNUH 2020-07-082) and conducted in accordance with the Declaration of Helsinki^37^. Informed consent was submitted by all subjects when they were enrolled.

## Results

### Characterization of immune features of COVID-19 patients related to the clinical severity

All samples were collected from Korean subjects with mild or severe-illness COVID-19 and healthy status. Their clinical characteristics and laboratory findings were summarized in Table 1. Clinically, only one MILD patient complained of fever, but over 85% of SEVERE patients had a fever at the time of blood sampling (*P* = 0.0101). There was no significant difference between MILD and SEVERE groups regarding underlying diseases. Contrary to needing mechanical ventilation for all SEVERE patients, all MILD patients remained stable without medical intervention during the isolation period except for some analgesics (*P* = 0.0002). The sampling point for this study was relative early, which was five to seven days after illness onset. In line with our previous report, C-reactive protein (P = 0.0003) and albumin (P = 0.0068) showed significant differences between the MILD and SEVERE groups^24^.

**Table 1:** Characteristics and laboratory findings of patients with COVID-19.

	Mild cases, n = 8	Severe cases, n = 7	*P-*value
**Characteristics**			
Age, years	51.3 (26–97)	62 (36–78)	0.1787
Male	6 (75)	4 (57)	0.6084
Body mass index, kg/m^2^	22.3 (11.8–26.6)	23.3 (20.3–27.7)	> 0.9999
Fever	1 (12.5)	6 (85.7)	0.0101
Sampling point from symptom onset (day)	6.9 (5–10)	7.6 (5–11)	0.5859
Mechanical ventilator use	0 (0)	7 (100)	0.0002
Modified Early Warning Score (MEWS)	1.3 (1–2)	2.6 (2–3)	0.0023
National Early Warning Score (NEWS)	0.1 (0–1)	5 (1–8)	0.0005
Sequential Organ Failure Assessment (SOFA) score	0.1 (0–1)	2.7 (0–6)	0.0039
**Underlying conditions**			
Cardiovascular disease	0 (0)	0 (0)	> 0.9999
Cerebrovascular disease	0 (0)	0 (0)	> 0.9999
Diabetes mellitus	0 (0)	1 (14.3)	0.4667
Chronic kidney disease	0 (0)	1 (14.3)	0.4667
Charlson Comorbidity Index (CCI)	1.3 (0–5)	3.1 (0–6)	0.0847
**Laboratory findings**			
White blood cell count, × 10^3^/mm^3^	4.3 (3.7–5.5)	6.3 (2.8–11.8)	0.267
Neutrophil, × 10^3^/mm^3^	2.8 (1.9–3.6)	4.7 (2.1–10.4)	0.1206
Lymphocyte, × 10^3^/mm^3^	1.1 (0.5–1.9)	1.1 (0.6–1.9)	0.8665
Neutrophil-to-lymphocyte ratio	3.1 (1.1–5.8)	4.8 (2.5–11.0)	0.1893
Monocyte, × 10^3^/mm^3^	0.4 (0.2–0.6)	0.5 (0.1–0.9)	0.4129
Monocyte, %	8.4 (5.8–13.0)	7.8 (3.1–14.3)	0.8665
Platelet, × 10^3^/mm^3^	192.8 (107–314)	187.7 (97–269)	> 0.9999
Alanine aminotransferase, U/L	37.8 (12–121)	23.9 (8–53)	0.9282
Aspartate aminotransferase, U/L	27.1 (13–50)	41.6 (19–110)	0.2922
Albumin, g/dL	4.1 (3.4–4.6)	3.0 (2.3–4.1)	0.0068
Total bilirubin, mg/dL	0.6 (0.1–1.9)	0.7 (0.1–2.27)	0.4834
Lactate dehydrogenase, U/L	404.4 (280–554)	701.3 (340–1461)	0.0541
C-reactive protein, mg/dL	0.7 (0.3–1.7)	8.1 (2.3–12.4)	0.0003

Data were presented as mean (ranges) or numbers (%). P-values were calculated by Mann–Whitney tests.

### Transcriptome analysis show that both *IL17RA* and *THBS1* gene levels are upregulated in severe COVID19 patients’ blood

**Figure 1:**
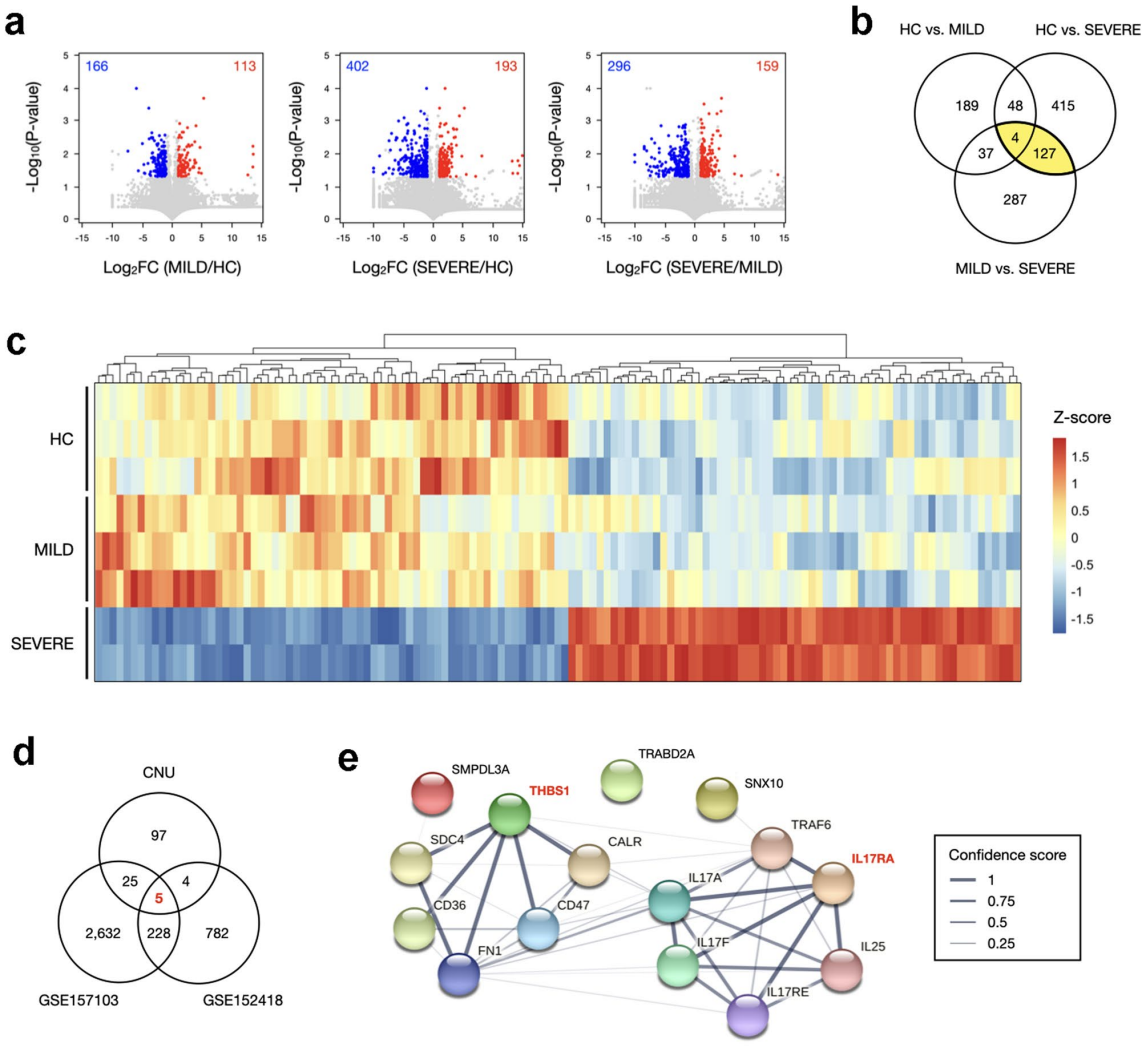
RNA-seq based transcriptome profiling of PBMCs from COVID-19 patients and healthy controls. (**a**) Volcano plots representing differentially expressed genes (DEGs) with the log2-fold change plotted against the negative log10 p-value for the three groups. Red and blue dots indicate significantly upregulated and downregulated genes, respectively. (**b**) Venn diagram showing the overlap of DEGs among each comparison. (**c**) A heatmap shows commonly identified 131 DEGs of HC versus SEVERE and MILD versus SEVERE. Expression levels are normalized to Z-score and cluster by Euclidean distance matrix. (**d**) Venn diagram showing the overlap of genes among each comparison of two publicly downloaded datasets and our own RNA-seq data. (**e**) Protein network analysis using the STRING database.

To explore gene expression patterns and identify significantly changed genes in severe COVID-19 cases, we generated RNA-Seq data of PBMCs from five COVID-19 patients, including two SEVERE and three MILD phenotypes and three HC. Three pairwise comparisons showed that overall transcriptome profiles did not appear to be very much different, even though the Spearman correlation coefficient (*ρ*) between HC and MILD (*ρ* = 0.966) was slightly higher than that between SEVERE and HCs (*ρ* = 0.951) and that between SEVERE and MILD (*ρ* = 0.947) (Supplementary Fig. 1a). Next, we identified DEGs for each dataset separately and their related pathways and functions by integrated bioinformatics analysis. Using 279 DEGs of HC versus MILD, 595 DEGs of SEVERE versus HC, and 455 DEGs of SEVERE versus MILD (Fig. 1a), gene ontology (GO) enrichment analysis was performed to investigate the main function of target genes. Although there were no significant enriched GO terms or pathways among 279 DEGs of HC versus MILD, the DEGs from the comparisons with SEVERE had enriched GO terms. 595 DEGs of SEVERE versus HC showed enrichment functionalities related to ribosome-related pathways, including SRP-dependent cotranslational protein targeting to membrane, rRNA processing, and translational initiation (Supplementary Fig. 1b). Intriguingly, we found a specific enrichment of genes associated with T cell activation and its signaling pathways among 455 DEGs of SEVERE versus MILD (Supplementary Fig. 1c). These findings convincingly indicated the alteration of expression of immune-related genes in the SEVERE COVID-19 patients.

To identify SEVERE-specific expression marker genes, we sorted out genes that were differentially expressed both between SEVERE versus HC and between SEVERE versus MILD. Of the 131 commonly altered DEGs in severe COVID-19 patients (Fig. 1b), 64 genes were upregulated, and 67 genes were downregulated in SEVERE compared to both MILD and HC (Fig. 1c).

Following that, we sought to validate our results using two publicly available human RNA-Seq datasets from PBMC samples among COIVD-19 patients. In the GSE157103 cohort, 2890 DEGs were identified between 42 severe and 58 moderate cases of COVID-19-positive patients (Supplementary Fig. 2a). In addition, 1019 DEGs were screened from the GSE152418 cohort, including 12 severe and 4 moderate COVID-19 patients (Supplementary Fig. 2b). As a result of comparing them with our experimental result, we found five putative SEVERE-specific expression marker genes, which were four upregulated genes (*IL17RA*, *SMPDL3A*, *SNX10*, and *THBS1*) and one downregulated gene (*TRABD2A*) (Fig. 1d). Furthermore, an investigation of protein–protein interaction using STRING database^30^ indicated that *IL17RA* and *THBS1* were characteristically shown as a hub and directly connected with several genes that are related to the cytokine and interleukin signaling pathways in the immune system (Fig. 1e). These data suggest that both *IL17RA* and *THBS1* levels are upregulated in PBMCs from severe COVID-19 patients compared to those from HC and mild patients.

### In the severe COVID-19 patients, *IL17RA *was mainly expressed in monocytes and neutrophils, whereas *THBS1* was highly expressed in platelets

**Figure 2:**
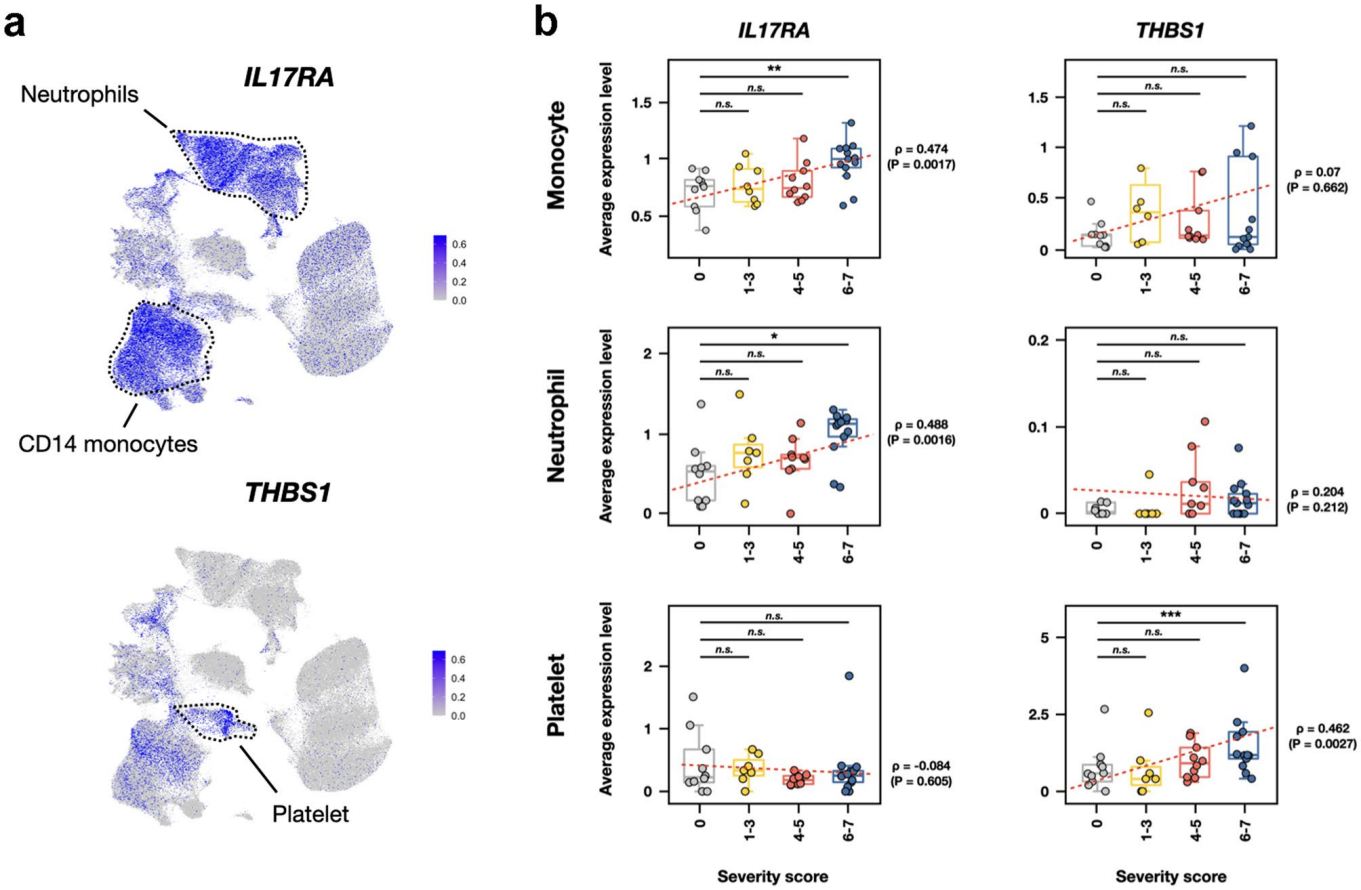
In severe and fatal COVID-19 patients, *IL17RA* expression is elevated in monocytes and neutrophils, but *THBS1* expression is elevated in platelets. (**a**) Two-dimensional UMAP projections of single cells from the GSE174072 cohort scRNA-Seq data colored by expression levels of *IL17RA* and *THBS1*. (**b**) Box plots depicting average expression levels of *IL17RA* and *THBS1* in monocyte (top), neutrophil (middle), and platelet (bottom). Patients are grouped by the severity score at the time of sample collection. *P < 0.05; **P < 0.01; ***P < 0.001; *n.s* not significant at *P* = 0.05 by two-sided Wilcoxon rank-sum test.

To investigate which immune-related cell types are responsive to *IL17RA* and *THBS1*, we used the GSE174072 cohort^34^ scRNA-Seq data consisting of over 175,000 single transcriptomes from 33 COVID-19 patients and 8 healthy controls with WHO COVID-19 severity scores. Given preprocessed scRNA-Seq data, we visualized them in two dimensions using the uniform manifold approximation and projection (UMAP) method and obtained the same plot as the original study (Supplementary Fig. 3). In the 14 distinct cell type clusters by UMAP plotting, *IL17RA* expression was most abundantly observed in ‘*CD14*-positive monocyte’ and ‘neutrophil’ cell types. On the other hand, *THBS1* expression showed distinctly different patterns, which was just a weak expression in ‘*CD14*-positive monocytes’ and no evident expression in ‘neutrophil’ cell types, but mainly observed in ‘platelets’ (Fig. 2a). We next questioned whether these gene expression patterns are predictive of COVID-19 severity scores. In general, the expression levels of *IL17RA* gene had significantly positive correlations with COVID-19 severity scores in *CD14*- positive monocytes (*ρ* = 0.474 and *P* = 0.0017) and neutrophils (*ρ* = 0.488 and *P* = 0.0016) (Fig. 2b, left panels). Notably, the *IL17RA* gene expression levels at severity score 6–7 were significantly higher than those at severity score 0 in *CD14*-positive monocyte (*P* = 0.0015, Wilcoxon rank-sum test) and neutrophil (*P* = 0.0100, Wilcoxon rank-sum test) cell types, whereas these changes were not observed in the *THBS1* gene expression patterns (Fig. 2b, right panels). In contrast, the expression levels of the *THBS1* gene were significantly (*P* = 0.0009, Wilcoxon rank-sum test) upregulated in the platelets from a severe group of COVID-19 patients. Altogether, these data strongly indicate that both monocytes and neutrophils are the major sources of *IL17RA*, whereas the platelets are the principal origin for *THBS1*, in human immune cell types. Moreover, elevated expression levels of *IL17RA* and *THBS1* in the severe COVID-19 patients suggest that those genes could serve as indicators of COVID-19 severity.

### *Hsa-miR-29a-3p* is a potential microRNA targeting *IL17RA* and *THBS1* in PBMCs from severe COVID-19 patients

**Figure 3:**
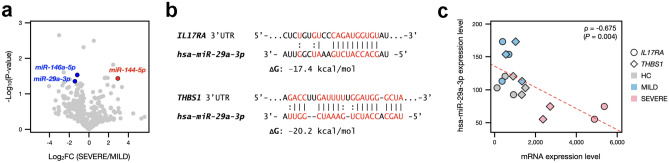
Correlation between the expression levels of *hsa-miR-29a-3p* and its two putative target genes in the severe COVID-19 samples. (**a**) Volcano plots representing differentially expressed miRNAs with the log2-fold change plotted against the negative log10 p-value of the compared groups. Red and blue dots indicate upregulated and downregulated miRNAs, respectively, in the severe COVID-19 samples. (**b**) Sequence alignments of *hsa-miR-29a-3p* with human *IL17RA* or *THBS1* mRNA 3′-UTRs. The prediction of binding sites and free energy (ΔG) were obtained from miRWalk 3.0 online prediction software. (**c**) Scatter plots showing the expression levels of *hsa-miR-29a-3p* versus *IL17RA* and *THBS1* in all eight subjects. *ρ* indicates Spearman’s correlation coefficient.

Recently, it has been convincingly demonstrated that host and viral-encoded miRNAs are essential for replication and infection of SARS-CoV-2^38–40^. Therefore, we sought to identify human miRNAs that were differentially expressed in severe COVID-19 samples and thus performed smRNA-seq experiments. After filtering lowly expressed miRNAs based on the threshold of normalized read counts < 5, two significantly downregulated (*hsa-miR-29a-3p* and *hsa-miR-146a-5p*) and one significantly upregulated (*hsa-miR-144-5p*) miRNAs were identified (Fig. 3a).

We questioned whether these miRNAs especially downregulated in severe COVID-19 samples were predicted to bind the 3′-UTR of *IL17RA* and *THBS1*. Analysis of the miRWalk database found that *hsa-miR-29a-3p* could bind to both *IL17RA* and *THBS1* (Fig. 3b). For the *IL17RA* mRNA, there were a total of 13 pairings beginning at the third position from the 5′-end of the *hsa-miR-29a-3p*, with two guanine-uracil wobble base pairs, given the theoretically calculated free energy of − 17.4 kcal/mol (Fig. 3b, upper panel). For the *THBS1* mRNA, the miRNA target sites lacked both perfect seed pairing and 3′-compensatory pairing but instead had 17 Watson–Crick pairs and three wobble pairs, giving the theoretically calculated free energy of − 20.2 kcal/mol (Fig. 3b, lower panel). Further, we found a significant negative correlation (*ρ* = − 0.675 and *P* = 0.004) between the expression levels of *hsa-miR-29a-3p* and two target genes (Fig. 3c). These findings implied that elevated *IL17RA* and *THBS1* mRNA expressions could be partly associated with dysregulation of *hsa-miR-29a-3p* in severe COVID-19 patients.

### *THBS1*, but not *IL17RA*, is elevated in PBMCs from COVID-19 patients and modulated by *hsa-miR-29a-3p*

**Figure 4:**
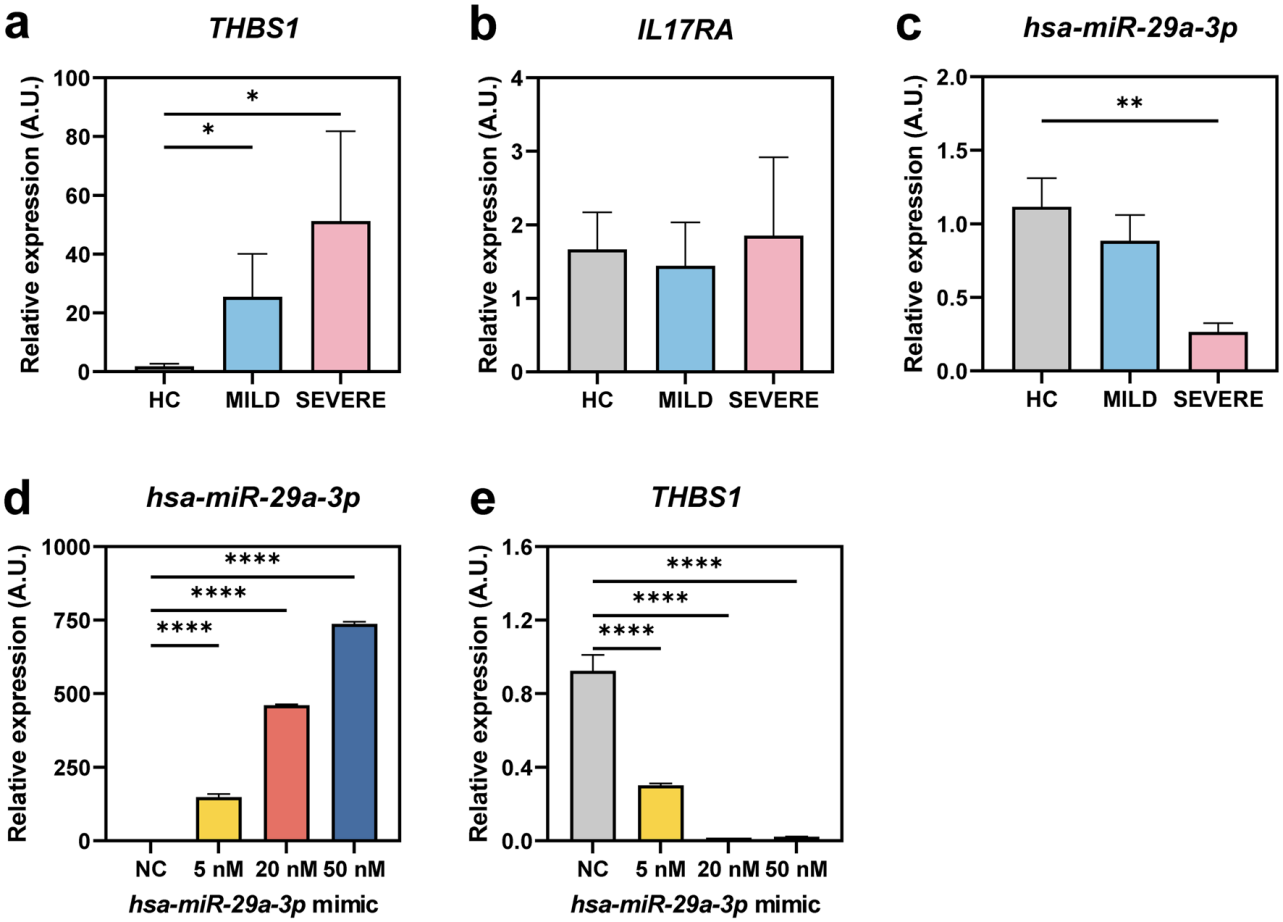
*THBS1* is upregulated, but *hsa-miR-29a-3p* is downregulated, in PBMCs from severe patients, and *THBS1* is targeted by *hsa-miR-29a-3p*. (**a**–**c**) Human PBMCs were isolated from mild (n = 8), severe (n = 7) patients and HCs (n = 8). *THBS1* (**a**), *IL17RA* (**b**), and *hsa-miR-29a-3p* (**c**) expression levels were measured by qRT-PCR analysis. (**d**,**e**) THP-1 cells were transfected with either negative control (50 nM) or *hsa-miR-29a-3p* mimic (5, 20, 50 nM). After transfection, total RNA was extracted for the measurement of *hsa-miR-29a-3p* (**d**) and *THBS1* (**e**) expression levels. Experiments were performed duplicate or triplicate and values are presented as means ± SEM (**a**–**e**). *P < 0.05; **P < 0.01; ***P < 0.001; ****P < 0.0001. Kruskal–Wallis test (**a**–**c**) and One-way ANOVA (**d**,**e**). *NC* negative control of *hsa-miR-29a-3p* mimic.

To further validate the bioinformatics results, we performed qRT-PCR analysis for *IL17RA* and *THBS1* in PBMC samples from COVID-19 patients (7 severe and 8 mild patients) and 8 healthy controls. We found that the mRNA expression of *THBS1*, but not *IL17RA*, was significantly increased in mild and severe patients compared to those from HCs (Fig. 4a,b). In addition, the *hsa-miR-29a-3p* level was significantly downregulated in PBMCs from severe patients compared with those from HCs (Fig. 4c). Because we found the upregulation of *THBS1* and downregulation of *hsa-miR-29a-3p* in severe patients, we further investigated the effects of *hsa-miR-29a-3p* upon the mRNA expression of *THBS1* in human THP-1 cells. As shown in Fig. 4d,e, we found that *hsa-miR-29a-3p* was overexpressed by *hsa-miR-29a-3p* mimic transfection, whereas *THBS1* mRNA expression was downregulated. Collectively, these data strongly suggest that *THBS1* is upregulated, whereas *hsa-miR-29a-3p* was downregulated, in PBMCs from severe patients. In addition, our data indicate that *hsa-miR-29a-3p* overexpression negatively regulated the *THBS1* level in human monocytic cells.

## Discussion

Recently, several studies have revealed that severe COVID-19 patients exhibit dysregulated immune responses during SARS-CoV-2 infection^14–16,41^. The lung pathology of SARS-CoV-2 infections represents diffuse pulmonary intravascular thrombosis associated with extensive inflammation^17,42,43^. The increased risk of venous thromboembolism is often shown to the upregulation of circulating D-dimer levels in COVID-19 patients^44^. There are close interactions between viruses and host factors involved in the immunothrombosis (coagulation and immune system). In addition, these crosstalks can be continued long after the clearance of viruses and influence the pathogenesis of different stages of COVID-19^17,45^. Recent studies with meta-analyses have proposed the promising effects of therapeutic heparin for moderately ill COVID-19 patients^46^. Indeed, heparin anticoagulation therapy leads to a decrease in the risk of mortality in hospitalized patients with COVID‐19^43,44^. However, it has not been widely known about the exact predicting markers for COVID-19 severity in the context of thromboembolism.

From the combinatorial analysis of messenger RNA-Seq data of three cohorts, this study discovered that *THBS1* was increased in severe COVID-19. The single-cell RNA-seq dataset revealed that *THBS1* was expressed in monocytes but had no significant change in monocytes with severity. On the other hand, we found that *THBS1* expression was mainly in platelets and high in severe COVID-19 patients. THBS1 is well known to be secreted by thrombin-activated platelets and is a significant player in thromboembolism^47–49^. Recent studies show that THBS1 is associated with not only thrombus formation but also immunomodulator^50,51^. In the immune and infection context, THBS1 and its receptor, CD47, inhibit T cell differentiation^52–54^. A previous proteomics study found significantly increased THBS1 levels in plasma from COVID-19 patients than healthy controls^55^. Although the sample size is small, we also confirmed that *THBS1* was upregulated in PBMCs from mild and severe patients, compared with those from HCs. Additional studies are warranted to examine the role of THBS1 in the disease progression and pathogenesis of COVID-19 and how it is upregulated during coagulation cascades and inflammatory responses.

MiRNAs have been recognized as potential biomarkers and regulators in the pathological responses during SARS-CoV2 infection^21–23^. Through targeting numerous genes involved in the pathophysiological responses during infection, the candidate miRNAs may participate in the modulation of a variety of molecular functions of target genes which influence the distinct patterns or the clinical outcomes of COVID-19^21,23^. Our data with the decreased expression of *hsa-miR-29a-3p* in severe patients partly correlate with previous findings that *hsa-miR-29a-3p* level is suppressed in the serum and plasma from severe group of COVID-19 patients^56,57^. Interestingly, the decreased level of *hsa-miR-29a-3p* is negatively associated with *COL5A3* in any grade of COVID-19 patients^56^. Our data is unique in showing the negative correlation of *hsa-miR-29a-3p* with *IL17RA* and *THBS1* in all subjects analyzed in the present study. Further experimental analysis strongly suggest that the reduced level of *hsa-miR-29a-3p* contributes to the excessive expression of *THBS1* in severe cases of COVID-19. To our knowledge, this is the first report to reveal that *THBS1* is potentially targeted by *hsa-miR-29a-3p*, which level is dysregulated in severe patients. A recent study also showed that miR-29a has an inhibitory function against different strains of influenza A infection by targeting the frizzled 5 receptor^58^. Furthermore, the miR-29a level showed an inverse correlation with HIV-1 replication and propagation^59,60^, suggesting that miR-29a plays an essential role in antiviral responses during viral infection. The recent in silico data that miR-29a has a high affinity to the SARS-CoV-2 genome^61^ highlight that miR-29a may be an attractive therapeutic target for SARS-CoV-2 infection. Future studies are urgently needed to evaluate whether *hsa-miR-29a-3p* contributes to antiviral and anti-inflammatory responses in the severe status of COVID-19.

In addition, our in silico analysis data showed that *IL17RA* as well as *THBS1* was predicted to be a target of *miR-29a-3p*. Th17 cells are the T lymphocyte subsets that mainly produce the cytokine IL-17A^62^. IL-17 is also produced by other cell types such as CD8+ T cells, γδ T cells, and natural killer cells^63^. Th17 cells can be divided into two types; host protective Th17 subset expressing IL-17 and IL-10^64,65^ and inflammatory Th17 cell type with the increased expression of IL-17, IL-22, and IFN-γ^66,67^. So far, it has been known that there are at least six IL-17 family members, IL-17A (usually called IL-17), IL-17B, IL-17C, IL-17D, IL-17E/IL-25, and IL-17F^68^. IL-17A and its receptor (IL17RA) are the best-characterized components that trigger downstream signaling pathways to activate pathologic inflammatory events^68,69^. IL-17/IL17RA signaling triggers the production of CXCL1, CXCL2, CXCL5, and CXCL8/IL-8, thereby inducing the recruitment of neutrophils^62^. This study repeatedly observed that IL17RA was consistently elevated in four different RNA-seq datasets profiling peripheral blood from severe COVID-19 patients, including a single-cell dataset. Emerging data suggest that the treatment with monoclonal antibodies targeting IL-17/IL17R (e.g., Ixekizumab, Secukinumab, and Brodalumab) is effective in various immune-mediated diseases^68^. It is largely unknown about the clinical significance of the IL17R levels to date. A recent finding by Scalia et al. showed that, in the serum of 35 Italian COVID-19 patients, IL-17A is higher, but the soluble IL17RA is lower in advanced severity. The increase of serum IL17RA prevents the interaction between IL-17 and its cell receptor, suggesting the benefit of monoclonal antibodies targeting the IL-17 pathway for COVID-19 treatment^70^. Therefore, it is warranted to accumulate more data to understand the clinical relevance of IL17RA in the context of severe COVID-19 treatment. In addition, a future study with a large cohort analysis should clarify whether IL17RA is the molecular target of *has-miR-29a-3p* in the context of severity during COVID-19.

Our work has various limitations, including a small sample size of Korean COVID-19 patients and a lack of gene expression validation in a larger population due to facility and patient enrollment constraints. Nevertheless, the increased *THBS1* and decreased *has-miR-29a-3p* in severely ill patients indicate that they are potentially valuable candidates for predicting clinical manifestations of COVID-19. Therefore, further studies in a large cohort are warranted to offer novel biomarkers and therapeutic options based on the *THBS1*, *IL17RA*, and *miR-29a-3p* for the treatment of severe COVID-19 patients.

## Supplementary Information

Supplementary Figures.

## Data Availability

All RNA-Seq data generated in this study are available in the NCBI Gene Expression Omnibus (GEO) through accession numbers SRR18361588-SRR18361603 under BioProject PRJNA817356.
