# Supplementary material for: Dysregulated thrombospondin 1 and miRNA-29a-3p in severe COVID-19
Source: Sci Rep. 2022 Dec 8;12:21227. doi: 10.1038/s41598-022-23533-x (PMC9732043; doi:10.1038/s41598-022-23533-x)
Supplement: Supplementary file 1 — Supplementary Figures. [file 41598_2022_23533_MOESM1_ESM.pdf]

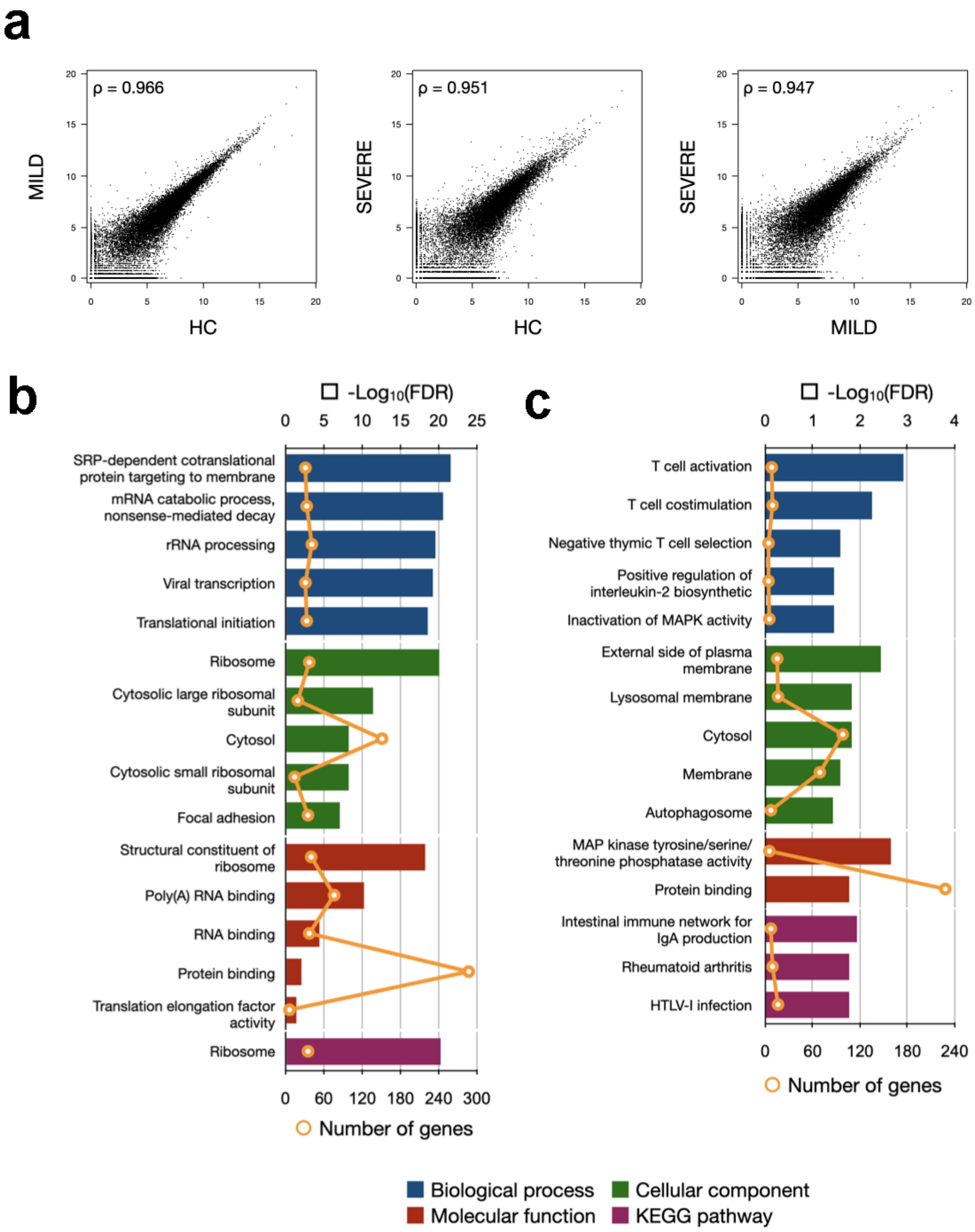

Supplementary Figure 1. Global transcriptome comparisons and gene ontology enrichment analysis. (**a**) Scatter plots representing global gene expression profiles with log2-transformed expression levels among three groups. ρ indicates Spearman's correlation coefficient. The top

five categories of gene ontology and KEGG pathway analysis were shown in panels (**b**) and (**c**) from comparisons HC versus SEVERE group and MILD versus SEVERE group, respectively.

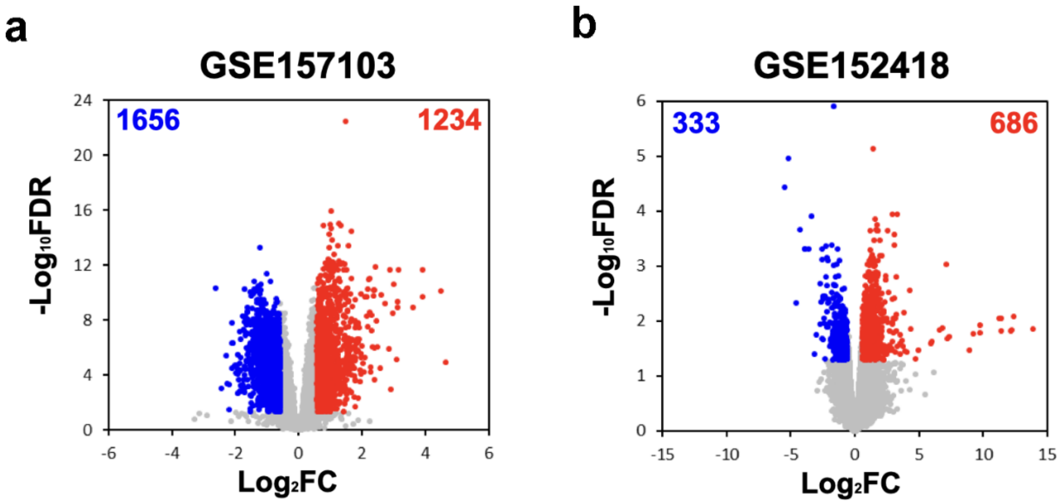

Supplementary Figure 2. Comparison of transcriptome data obtained from two publicly available RNA-seq datasets. Volcano plots representing whole transcriptomes with the log2-fold change plotted against the negative log10 adjusted P-value (FDR) from (**a**) GSE157103 COVID-19 cohort and (**b**) GSE152418 COVID-19 cohort. Red and blue dots represent significantly up- and down-regulated genes in severe COVID-19 patient samples. Grey dots indicate genes that are altered insignificantly.

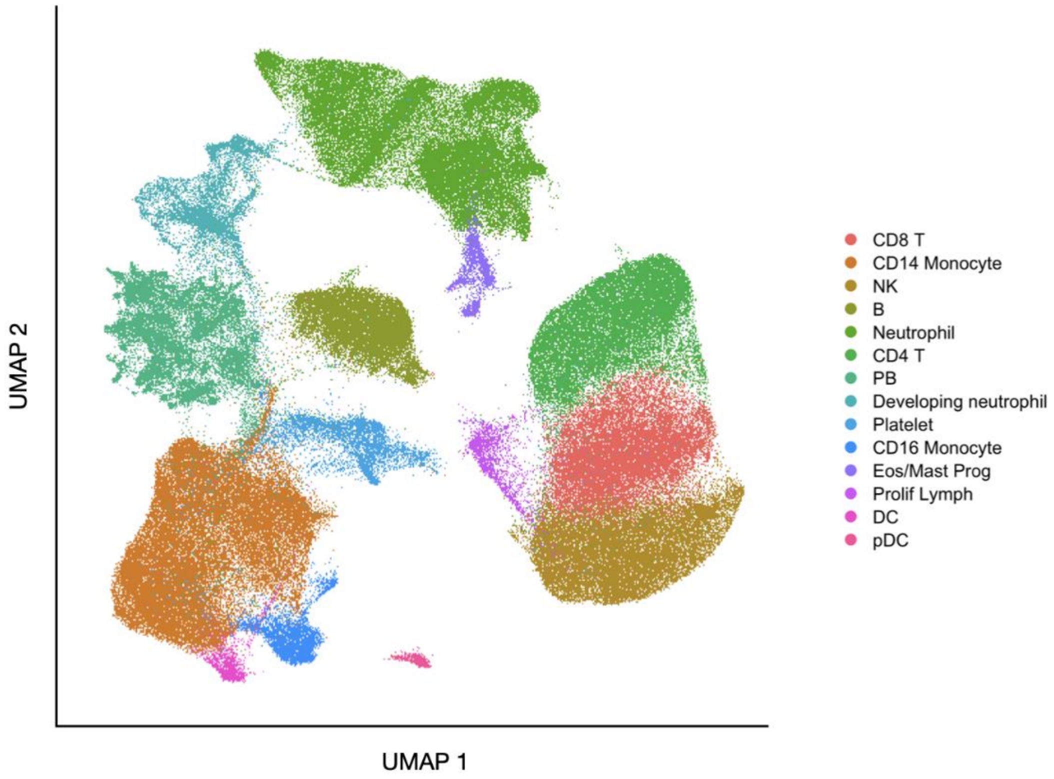

Supplementary Figure 3. Two-dimensional UMAP projections of single cells from the GSE174072 cohort scRNA-Seq data
